# Supplementary material for: The Protective Role of KANK1 in Podocyte Injury
Source: Int J Mol Sci. 2024 May 27;25(11):5808. doi: 10.3390/ijms25115808 (PMC11172089; doi:10.3390/ijms25115808)
Supplement: Supplementary file 1 [file ijms-25-05808-s001.zip › ijms-3004922-supplementary.pdf]

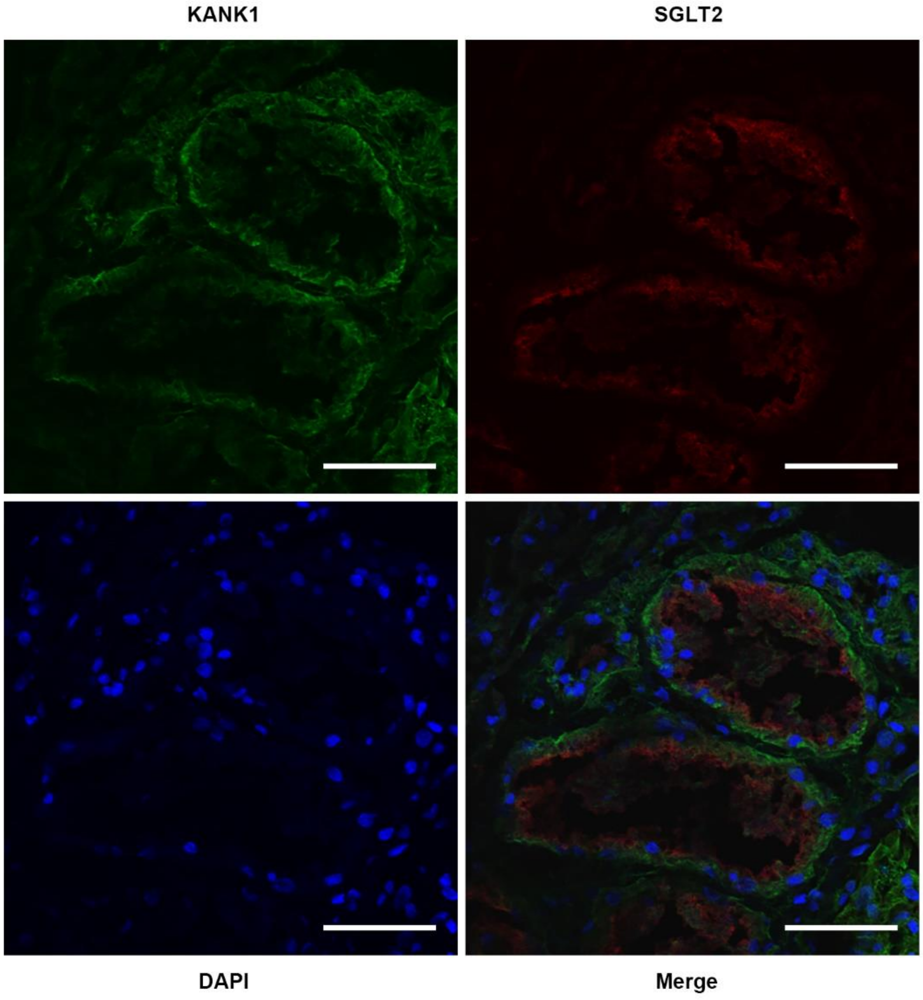

Supplementary Figure S1. KANK1 expression in the proximal tubular basement membrane of the human kidneys. In human renal tubules, KANK1 was extensively localized within the proximal tubular basement membranes, and it did not exhibit colocalization with sodium-glucose transporter 2 (SGLT2), which was predominantly expressed along the brush borders of the proximal tubules. DAPI, 4',6-diamidino-2-phenylindole. Scale bars indicate 50 μm.

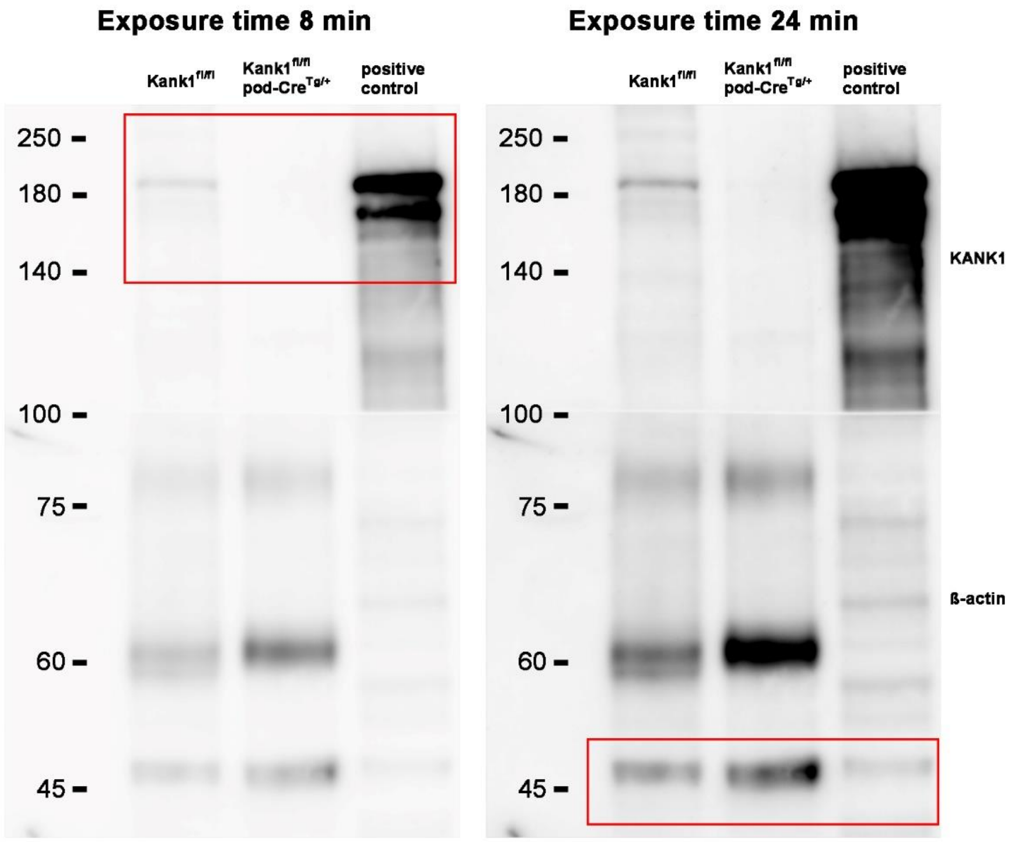

Supplementary Figure S2. Full-length results of Western blotting.

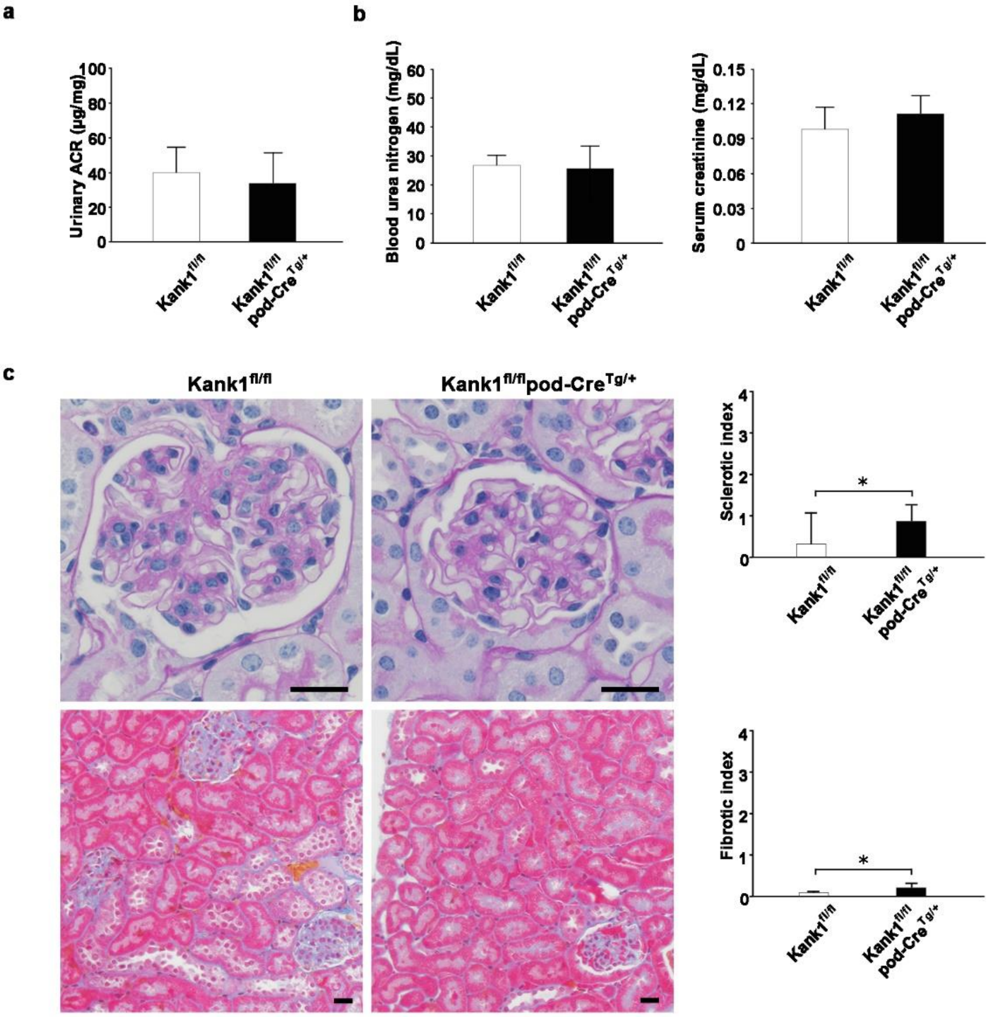

Supplementary Figure S3. Inactivation of Kank1 in podocytes at the age of six months did not result in albuminuria. (a) The urinary albumin-creatinine ratio (ACR) at six months old was not higher in the Kank1$^{fl/fl}$pod-Cre$^{Tg/+}$ group than in the Kank1$^{fl/fl}$ group. (b) There was no significant difference in the blood urea nitrogen (BUN) or serum creatinine (Cr) levels between the Kank1$^{fl/fl}$ group and the Kank1$^{fl/fl}$pod-Cre$^{Tg/+}$ group at six months old. (c) Sclerotic and fibrotic indices at six months old were significantly higher in the Kank1$^{fl/fl}$pod-Cre$^{Tg/+}$ group than in the Kank1$^{fl/fl}$ group (*P <0.05, respectively). Scale bars indicate 20 μm.

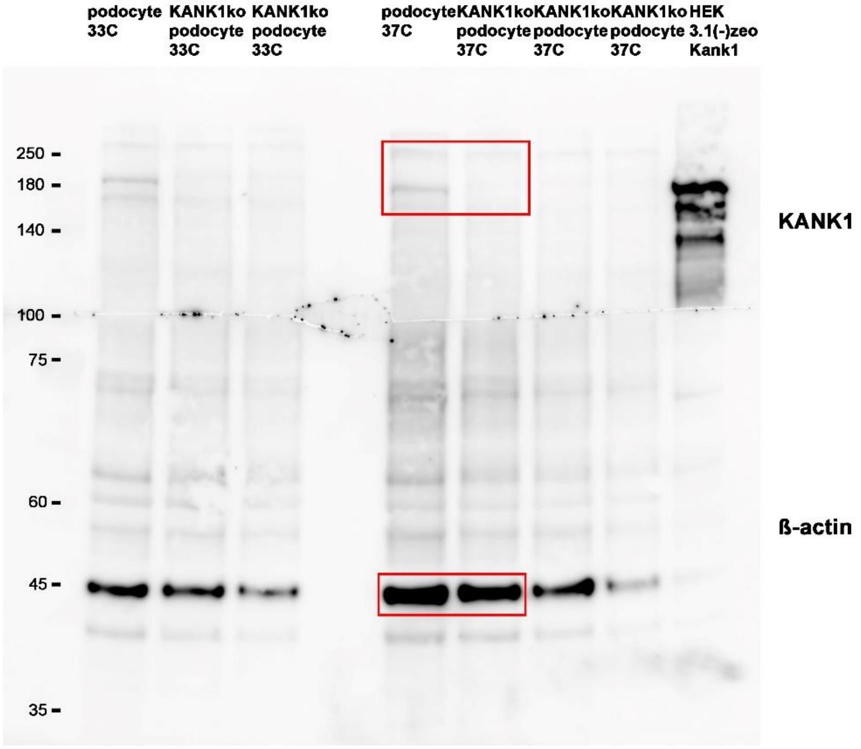

Supplementary Figure S4. Full-length results of Western blotting.

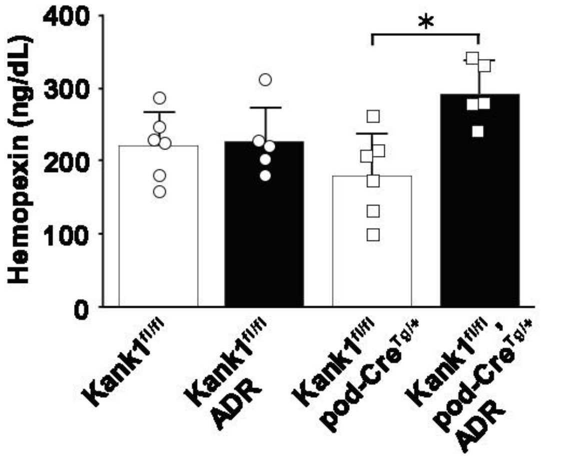

Supplementary Figure S5. The serum hemopexin levels before and after adriamycin (ADR) treatment. The serum hemopexin levels were significantly elevated in the Kank1$^{fl/fl}$pod-Cre$^{Tg/+}$ mice after ADR treatment, while there was no significant difference between serum hemopexin levels in the Kank1$^{fl/fl}$ mice after ADR treatment.
